# Development of Enhanced Separation Techniques for Oligonucleotides Utilizing Mixed‐Mode Chromatography and 2D‐LC/UV/MS Analysis

**DOI:** 10.1002/jssc.70238

**Published:** 2025-08-05

**Authors:** Jakob Haglöf, Jenny M. Nilsson, Jufang Wu Ludvigsson

**Affiliations:** ^1^ Department of Medicinal Chemistry, Uppsala Biomedical Centre Uppsala University Uppsala Sweden; ^2^ Global Pharmaceutical Development, Pharmaceutical Technology & Development Operations, AstraZeneca Gothenburg Sweden

**Keywords:** 2D liquid chromatography, mass spectrometry, mixed‐mode chromatography, oligonucleotide, reversed‐phase/weak anion‐exchange

## Abstract

Oligonucleotides are traditionally analyzed using ion‐pair reversed‐phase liquid chromatography. In this project, we explored an alternative approach utilizing a reversed phase‐weak anion exchange mixed‐mode column, which combines separation based on both charge and hydrophobicity. Our findings indicate that the mixed‐mode column provided stronger retention and superior separation compared to the C18 column, eliminating the need for ion‐pairing reagents. Additionally, selectivity can be adjusted using gradient pH and buffer concentration as well as acetonitrile content. However, the use of a phosphate buffer was necessary to ensure adequate retention and separation, despite its incompatibility with MS detection. To address this issue, a 2DLC‐UV‐qTOF setup with multiple loops was established, where the second‐dimension LC, connected to the MS detector, required only an MS‐compatible mobile phase. Using a HILIC‐type LUNA Omega Sugar column in the second‐dimension LC, the MS intensity of the heart‐cut from the first dimension was increased 100‐fold, allowing for the characterization of the main peak.

## 1 Introduction

Oligonucleotides, short sequences of nucleic acids, play critical roles in biotechnology, medicine, and research. They are widely used as probes, primers, antisense agents, aptamers, and therapeutics [1, 2]. However, their complex structures, diverse chemical modifications, and high polarity pose significant challenges for analytical methods, particularly in achieving high resolution, sensitivity, and selectivity for impurity analysis [3].

Liquid chromatography (LC) is a widely adopted technique for oligonucleotide analysis, offering separation based on properties such as size, charge, hydrophobicity, and affinity. Among conventional LC methods, ion‐pair reversed‐phase liquid chromatography (IP‐RPLC) is often described as the gold standard for oligonucleotide analysis [4, 5, 6]. While widely used, and extensively researched, IP‐RPLC relies on ion‐pairing reagents, which are often incompatible with mass spectrometry (MS) detection, limiting its applicability [7, 8]. Other approaches for oligonucleotide analysis, including facilitating MS hyphenation, involve e.g. non‐ion pairing reversed‐phase (RP) chromatography with ammonium bicarbonate buffers [9], anion‐exchange chromatography (AEX) [10], size‐exclusion chromatography (SEC) [11], and HILIC [12], but all come with different limitations in resolution, peak shape, or recovery.

To address these challenges, mixed‐mode liquid chromatography (MMC) [13] has emerged as a promising alternative. MMC integrates two or more interaction modes, such as RP and ion‐exchange (IEC), RP and SEC, or IEC and SEC properties within a single column. This approach provides multiple selectivity factors and separation mechanisms, enabling enhanced resolution, improved peak shapes, and higher recovery rates, i.e., allowing more alternatives for method development [14].

The versatility of MMC lies in its ability to analyze oligonucleotides with diverse properties and modifications [15]. For instance, RP‐IEC chromatography is ideal for small‐to‐medium‐sized oligonucleotides with modifications such as phosphorothioates, methylphosphonates, and locked nucleic acids. RP‐SEC chromatography is better suited for large oligonucleotides with high molecular weight and low charge density, such as plasmids, ribozymes, and siRNAs. IEC‐SEC chromatography is relevant for separating oligonucleotides based on distinct charge states and sizes, such as triplex‐forming oligonucleotides, aptamers, and peptide nucleic acids. Nevertheless, MMC separations suffer from the same drawback as IEC, namely the high buffer ion concentrations needed in the mobile phase for analyte elution, making hyphenation to ESI‐MS detection challenging [16].

Another approach to overcome limitations of different modes of chromatography for oligonucleotide analysis is using two‐dimensional liquid chromatography (2DLC) [17, 18]. By utilizing different stationary phases in the first (1D) and second dimension (2D), or different mobile phases with the same stationary phase, enhanced selectivity and/or MS compatibility can be attained. Several different papers discuss the applicability of 2DLC for the analysis of oligonucleotides, e.g., Li et al. [19] using a HILIC 1D and IP‐RP 2D with ESI‐MS detection for analysis of 2–10 nt oligonucleotides; Roussis et al. [20] used an IEC‐IP‐RP system for therapeutic oligonucleotide impurity characterization; Stoll et al. [21] used IP‐RP in both dimensions, but with different mobile phases, while Kazarian et al. [22] used an AEX 1D, and hydrophobic interaction chromatography (HIC) 2D. Li et al. [16] used MMC for the 1D, followed by a 2D de‐salting setup for ESI‐MS compatibility. The same group [23] later developed a chiral × RP 2DLC method for enhanced separation power of phosphorothioate oligonucleotide diastereomers.

In this study, we explored an alternative approach leveraging a WAX‐RP mixed‐mode column that combines separation based on both charge and hydrophobicity in the 1D. However, achieving adequate retention and separation required the use of a phosphate buffer, which is incompatible with MS detection. To overcome this limitation, we implemented a 2DLC workflow with UV/MS detection. In this setup, the 2DLC, interfaced with the MS detector, utilized an MS‐compatible mobile phase. By employing a HILIC column developed for carbohydrate analysis in the 2D, the MS signal intensity for heart‐cuts from the 1D was amplified by 100‐fold, enabling detailed characterization of the peaks.

## 2 Materials and Methods

### 2.1 Chemicals and Materials

A 16 nucleotide long oligonucleotide with sequence GGG AAA mCmCmC TTT GAmCT was synthesized in‐house at AstraZeneca. Following synthesis, the oligonucleotide solution was de‐salted, but without further purification to contain relevant impurities for method development purposes. Oligonucleotide samples ranging from 136 to 1.36 µM were prepared in Milli‐Q water. A degraded oligonucleotide sample was prepared by exposing a 136 µM oligonucleotide sample for daylight and room temperatures for 6 months.

LC‐MS grade ACN was bought from Fisher Scientific (Hampton, NH, USA), 85% ortho‐phosphoric acid from Merck (Darmstadt, Germany), ammonium hydroxide for LC‐MS and ammonium bicarbonate (≥ 99%) from Sigma‐Aldrich (St. Louis, MO, USA), and Acetic acid of glacial analytical grade from Sharlau (Barcelona, Spain). Water was purified using a Milli‐Q‐system from Millipore (Billerica, MA, USA).

The stationary phases employed were an Acquity BEH C18 column, 2.1 mm × 50 mm, with 1.7 µM particles, and an Acquity BEH Amide column, 2.1 mm × 50 mm, with 1.7 µM particles, from Waters (Milford, MA, USA), an Acclaim Mixed‐Mode RP/WAX‐1 column, 3.0 mm × 50 mm, with 3 µM particles, from Thermo Scientific (Sunnyvale, CA, USA) and a LUNA Omega Sugar column, 2.1 mm × 50 mm, with 3 µM particles, from Phenomenex (Torrance, CA, USA).

### 2.2 Instrumentation and Conditions

The chromatography system for 1D analyses was an Acquity UPLC system from Waters (Milford, MA, USA), equipped with a flow‐through needle (FTN) sample manager (SM), binary solvent manager (BSM), and photodiode array (PDA) detector connected to a Synapt G2S qTOF mass spectrometer from Waters (Milford, MA, USA). For 2D chromatography the 1D system was connected to an Acquity column manager (CM) with three external valves and an additional BSM pump, all from Waters (Milford, MA, USA).

**FIGURE 1:**
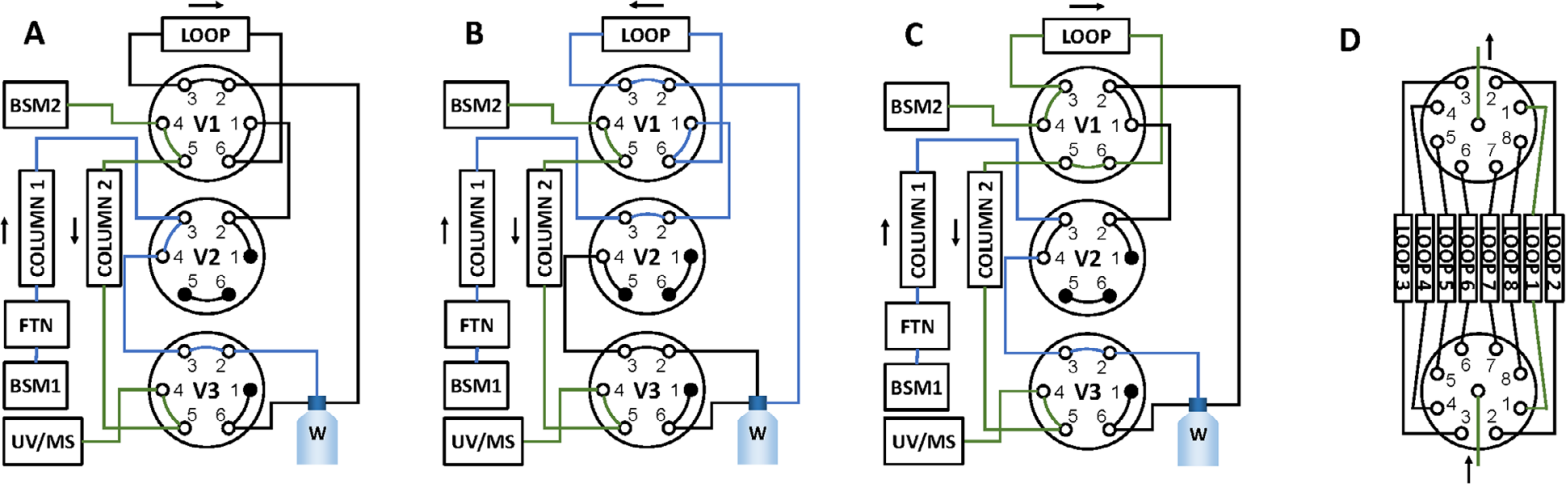
Illustration of 2DLC setup, with focus on the different valve's positions. (A) ^1^D‐separation of oligonucleotide sample, (B) heart cut of ^1^D eluent and collection in loop, (C) injection of stored loop volume onto the ^2^D‐system. Finally, (D) shows a schematic of the multi‐loop setup.

2D experiments used the chromatography setup schematically illustrated in Figure 1. Blue and green lines denote mobile phase flow for ^1^D separation and ^2^D separation, respectively. Figure 1A illustrates the ^1^D separation where the sample is injected onto the MMC stationary phase and the eluent goes to waste. In Figure 1B valve V2 has switched to the loop storage configuration, and the eluent from the ^1^D separation connects to the loop. Finally, in Figure 1C, valve V1 has switched to the ^2^D separation, injecting the stored volume in the loop onto the second stationary phase for separation of phosphate and oligonucleotide separation. In addition, in Figure 1C, the valve V3 is set to run eluent from the ^2^D separation to the detectors, however, the method incorporated a not shown 1‐min delay time to allow for the ^2^D eluent void volume to be routed to waste, before the eluted oligonucleotides can be detected using the UV and MS detectors.

The setup was further developed using an Acquity CM in place of a single loop to accommodate multiple loops of varying sizes (5–250 µL), see Figure 1D. The CM consists of two 9‐port valves, of which eight are used for pairwise connection of the loops, and the remaining ports, one on each valve, connected to the V1 valve. Depending on application, loops of different or similar sizes can be incorporated. Both external valves and loop selection can then be controlled and programmed in the MassLynx software (Waters, Milford, MA, USA), making the setup confined to a single method. For optimization, the ^1^D method was run in UV detection mode, and the timing for heart‐cuts and subsequent ^2^D analysis derived from it.

### 2.3 Optimized Final Methods

Mobile phase A for ^1^D analysis consisted of 70% 100 mM ammonium phosphate buffer pH 7 and 30% ACN, while mobile phase B consisted of 70% 200 mM ammonium phosphate buffer pH 8 and 30% ACN. The buffers were prepared by adding appropriate amounts of ammonium hydroxide to 100 and 200 mM phosphoric acid solutions for mobile phase A and B respectively. The final method uses a gradient from 40% B to 100% B over 25 min, with 5 min for reconditioning the column.

The mobile phases for ^2^D analysis consisted of Milli‐Q water and ACN in a 95:5 ratio for mobile phase A and a 5:95 ratio for mobile phase B. A linear gradient from 30% to 80 % A, i.e., 66.5% to 19% ACN, over 15 min was employed for the final method.

The flow rates for both ^1^D and ^2^D analyses were set to 0.3 mL/min, the PDA detector operated at 258 nm and the samples were kept at 8°C in the SM during analyses.

For ^2^D analyses, the valves were set to switch between different modes according to Figure 1A–C. Furthermore, loop sizes were determined using the ^1^D method in UV detection mode, via evaluation of retention time, peak volume, and resolution.

The Synapt G2S mass spectrometer was operated in negative ESI‐MS mode over a 500–2500 *m*/*z* range. The capillary voltage was set to 2.60 kV, the sample cone temperature to 40°C, the cone gas flow to 100 L/h and the source offset to 50 V. The source temperature was 120°C, the desolvation gas temperature 650°C, desolvation gas flow 500 L/h and nebulizer gas flow 7.0 bar. Calibration was performed in negative mode for the 500−2500 *m*/*z* range using sodium iodide. Lockspray with leucine enkephaline (554.246 *m*/*z*) was used as internal mass calibration for all analyses.

## 3 Results and Discussion

Building upon recent development in the use of mixed‐mode stationary phases for the characterization of oligonucleotides and related impurities, the Acclaim Mixed‐Mode RP/WAX‐1 column combines weak anion exchange and reversed phase properties. Compared with conventional anion exchange phases, the WAX structures allow for the modification of retention properties of the stationary phase with buffer pH in addition to charge‐related changes for the analyte, resulting in a more versatile, although more complex, retention properties profile. The stationary phase is marketed as a mixed RP‐IEC stationary phase aimed at a wide range of analytes, including organic and hydrophobic acids, peptides, vitamins, and pharmaceuticals in general. The stationary phase consists of long alkyl chains ending in amides or tertiary amines, and the selectivity is mainly influenced by pH, buffer ionic strength, and organic modifier content. The mixed‐mode properties together with a wide range of suitable mobile phase conditions for these variables make the stationary phase interesting for oligonucleotide analysis in addition to the marketed intended uses above.

### 3.1 1D RP‐WAX MMC Method Development

For analysis using IEC stationary phases, including MMCs encompassing IEC functionality, the oligonucleotide charges are in focus for method development. The main source for oligonucleotide charge is the phosphate groups in the backbone. With a pKa of ∼1 of the phosphate group, oligonucleotides have a negative unit charge per nucleotide in most mobile phase pH, i.e., above pH 3 [24]. Another source for oligonucleotide charge is the bases, with pKa for the corresponding acids between 2.4 and 4.6 depending on base [24], adding a positive charge to these bases at low acid pH. In addition, guanosine can display acidic properties at alkaline pH (pKa ∼9.5), allowing for additional negative charge at elevated pH. In practice IECs and MMCs with IEC properties often employ mobile phase buffers of pH 6−8 for oligonucleotide analysis, which essentially eliminate the possibilities of positive charges of the oligonucleotide bases and results in a more or less uniform unit negative charge per nucleotide for the sequence. Thus, alterations of mobile phase pH mainly influence the separation properties of the weak anion stationary phases rather than the analyzed oligonucleotide. However, this does not take into account common oligonucleotide impurities, including deaminations and depurinations, which for guanosine, with an acidic pKa just above 9 [24], might affect the oligonucleotide net charge, that can be utilized for enhanced separation selectivity [2].

The present investigation into the separation properties of the Acclaim Mixed‐Mode RP/WAX‐1 stationary phase included buffer type, ionic strength, pH, ACN composition, and temperature. The pKa of the WAX component in the stationary phase is not defined from the manufacturer, neither is the precise structure of the alkyl chains. The information provided is that the stationary phase consists of long straight alkyl chains, reminiscent of RP stationary phases, but ending in a tertiary amine. These generally exhibit dissociation constants in the pH 9−10 range, although heavily dependent on the residual chain lengths and branching [25]. Increased pH decreases the amine charge and thus the IEC‐based retention of the oligonucleotides. For the stationary phase employed here, the best selectivity could be found in the pH 6−8 interval, and a pH gradient from 7 to 8 were implemented to take full advantage of this for impurity analysis.

Besides mobile phase pH, buffer concentration heavily influences the retention of IEC stationary phases, and the Acclaim MMC stationary phase displays the same behavior. The high charge state of the oligonucleotides at pH 6–8 results in a strong interaction between oligonucleotide and stationary phase amines, and consequently high buffer concentrations in the order of 100–200 mM are required to elute the oligonucleotides. For higher selectivity, a buffer concentration gradient can be incorporated together with the pH gradient.

**FIGURE 2:**
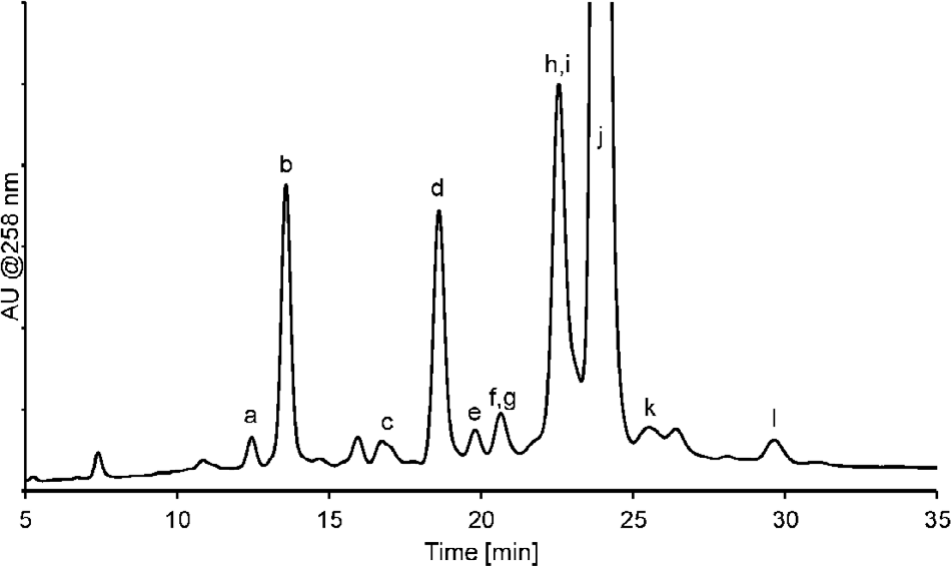
Analysis of degraded oligonucleotide sample on the Acclaim Mixed‐Mode RP/WAX‐1 column with an ammonium phosphate mobile phase buffer and 30% ACN with a pH and buffer concentration linear gradient from pH 7 (100 mM) to pH 8 (200 mM).

Figure 2 shows the resulting chromatogram from a degraded oligonucleotide sample in order to illustrate the separation properties of the method using constant 30% ACN mobile phase content with a combined pH (from pH 7 to 8) and buffer concentration (from 100 to 200 mM) 30 min gradient. The main oligonucleotide elutes after 24 min, with impurities eluting from 7 min (shorter varieties) to 30 min (longer varieties). The retention pattern is mainly based on oligonucleotide length (n nucleotides), but degradation products with depurinated nucleobases (e.g., peaks a and i) or degraded nucleosides (e.g., peaks e, f, h, and k) are also separated.

**FIGURE 3:**
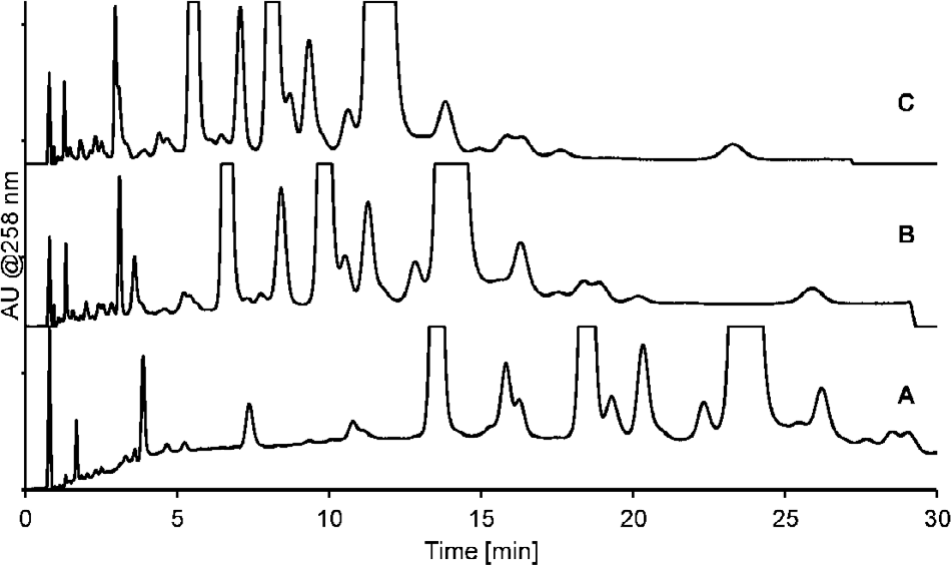
Analysis of oligonucleotide sample with impurities on the Acclaim Mixed‐Mode RP/WAX‐1 column with an ammonium phosphate mobile phase buffer and 30% ACN and pH and buffer concentration gradient from pH 7 (100 mM) to pH 8 (200 mM). Chromatograms illustrate different gradient settings with (A) 40%−100% B; (B) 80%−100 % B; and (C) 90%−100% B.

In this study the main oligonucleotide is 16 nucleotides long, with impurities ranging from 18 nucleotides and down. The combined pH and buffer concentration gradient can be adapted to allow for the separation of longer sequences as well, as shown in Figure 3. However, interestingly even with the same mobile phase A and B composition, selectivity is altered for certain sets of impurities, e.g., note retention changes for impurities at 3.9 and 7.4 min (Figure 3C), respectively. Therefore, care needs to be taken when adjusting the method gradient to other samples, but also allows for the option to change method selectivity based on the analysis needs.

**FIGURE 4:**
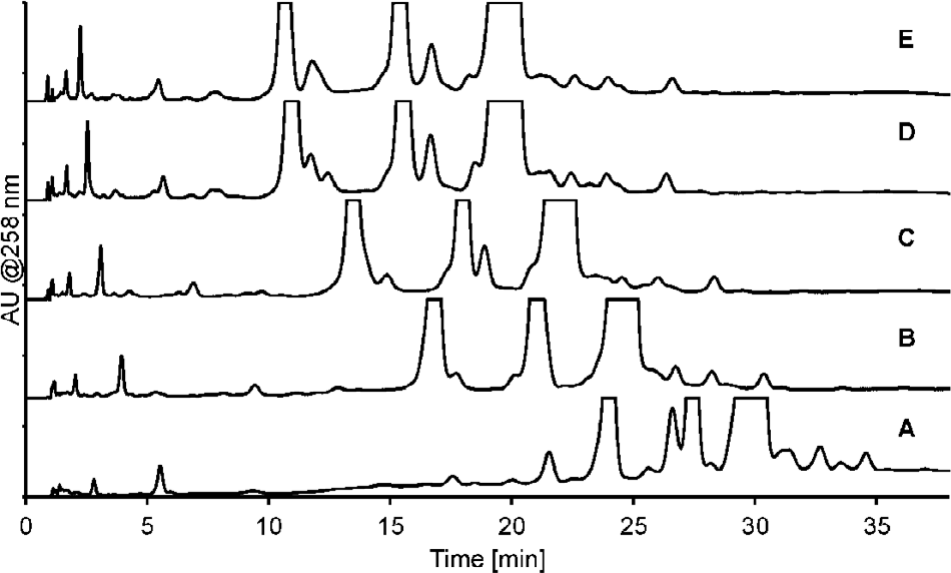
Analysis of oligonucleotide sample with impurities on the Acclaim Mixed‐Mode RP/WAX‐1 column with an ammonium phosphate mobile phase buffer and 15% (A, bottom) to 35% (E, top) ACN in 5% increments; pH and buffer concentration gradient from pH 7 (100 mM) to pH 8 (200 mM).

The methods employed in Figures 2 and 3 both utilize ammonium phosphate buffers combined with 30% ACN organic solvent additive. The buffer type and the ACN content has generally limited effect on the selectivity, but influences retention to a large degree, as exemplified for ACN content in Figure 4.

**FIGURE 5:**
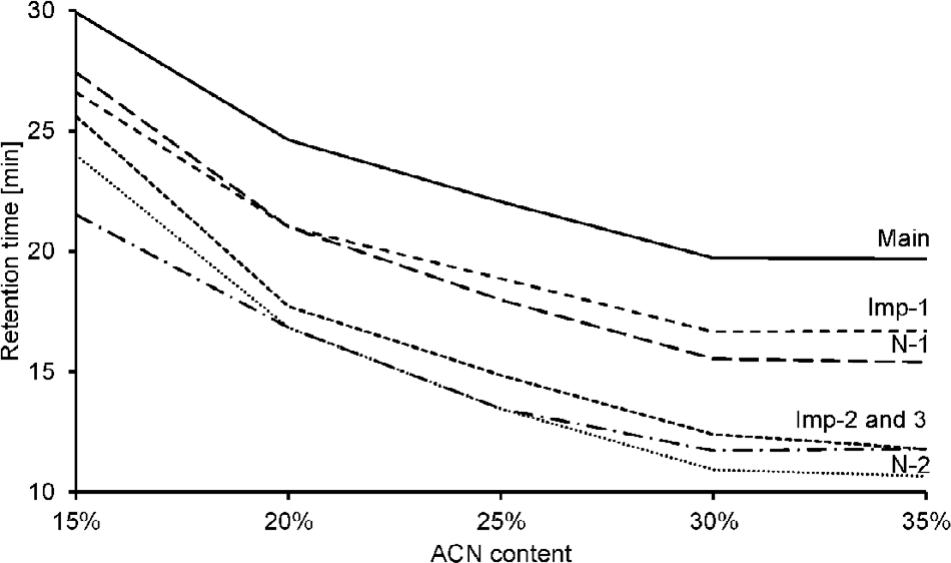
Changes in retention time for main oligonucleotide and selected impurities as a function of ACN content in the mobile phase.

However, depending on impurity properties, ACN content can influence selectivity as well, e.g. in Figure 4A the peak at 26.5 min in the bottom chromatogram, corresponding to 15% ACN, shows differential selectivity compared with the short‐mer peak at 27.5 min. When ACN content increases the retention decreases more for the degraded nucleoside than the short‐mer, illustrating that for individual impurities, the ACN content can change the IEC to RP retention contribution ratio. Ordonez et al. [26] discusses the retention characteristics for mixed mode stationary phases, and argues that the retention mechanisms of these phases are more complex than simple additive contributions and that changes in separation parameters often show non‐linear changes in retention profile, which aligns well with what was observed here. Figure 5 shows a graph of the retention as a function of mobile phase ACN content for selected impurities in the oligonucleotide sample, illustrating the impact on selectivity between impurities.

**FIGURE 6:**
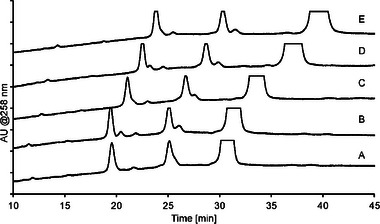
Analysis of oligonucleotide sample with impurities on the Acclaim Mixed‐Mode RP/WAX‐1 column at different temperatures from 30°C (A) to 50°C (E) in 5°C increments. The mobile phase comprised an ammonium phosphate mobile phase buffer and 20% ACN with a pH and buffer concentration linear gradient from pH 7 (100 mM) to pH 8 (200 mM).

In the end mobile phase ACN content mainly influenced the overall retention of both the main oligonucleotide and impurities and could be used to fine‐tune retention and overall analysis time with only limited effects on selectivity, thus complementing changes to buffer pH or ionic strength for method development purposes. Similarly, the gradient slope also affected the retention and hence the resolution of the separation, as illustrated in Figure 3. In addition to gradient and ACN content, column temperature also influenced the retention, with lower temperature decreasing overall retention, see Figure 6. Column temperatures from 25°C to 55°C were investigated, and a final temperature of 30°C was best suited for the analyses.

As discussed above, a mobile phase pH gradient from 7 to 8 resulted in the best selectivity. At this pH the choice of suitable buffers is limited. The vendor suggests phosphate buffers as the primary choice, but there are several associated issues with this buffer. Phosphate buffers are characterized by low volatility, making them a poor choice for MS hyphenation. In addition, phosphate buffers have poor solubility in several organic solvents, including ACN, which might limit the available range of method development if increased ACN content would be necessary for another sample set than the analysis demonstrated in Figure 2 with a mobile phase consisting of ACN and ammonium phosphate buffer. Therefore, several different buffer alternatives were investigated, including ammonium acetate, ammonium formate, and ammonium bicarbonate. However, all these buffers resulted in severely increased retention that could barely be accommodated using even higher buffer concentrations or acetonitrile content. Thus, it is evident that the presence of phosphate in the mobile phase is a necessity to achieve reasonable retention for oligonucleotide analysis on the Acclaim Mixed‐Mode RP/WAX‐1 stationary phase. The phosphates are more potent counter‐ions for the IEC part of the MMC stationary phase, showing a higher degree of competition with the oligonucleotides for the binding sites, possibly due to the higher negative charge of phosphate ions at pH 7−8. Acetate or formate ions have a single charge when dissociated; bicarbonate is di‐acidic, but the second pKa is 10.3 making the monocharged carbonate ion the most prevalent at pH 7−8.

The developed method shows promising results for oligonucleotide impurity analysis when combined with UV‐detection. Impurities of both length/size and charge, including common short‐ and long‐mers, depurination and deamination impurities, can be successfully separated from the main oligonucleotide as well as other impurities. The gradient can be adapted to samples with both longer or shorter oligonucleotides as needed, without the need for changes to the mobile phase composition.

However, due to the mobile phase composition with high concentrations of phosphate buffer, the method is not compatible with MS detection. The efforts to adapt the method for MS detection through the exchange of buffer type and/or changes in buffer concentrations showed limited results. Therefore, the developed method was considered for 2DLC adaptation, with enhanced selectivity and MS hyphenation.

### 3.2 2D Method Development

LC coupled to MS detection is a staple in impurity analysis, allowing for the unambiguous identification of separated components in a sample. The developed ^1^D method discussed above was adapted to 2DLC using an in‐house built 2DLC system based on the Waters Acquity UPLC, employing a set of eight loops of varying sizes. The system was coupled to serial in‐line UV and MS detectors, allowing for the combined detection of UV and MS signals.

The resulting 2DLC method was set‐up to heart cut a single chromatographic peak from the ^1^D method and store the cut volume in a pre‐defined loop. The loop volumes were chosen to allow for saving whole peaks eluting late in the ^1^D analysis (large volumes, up to 250 µL), as well as partial peaks eluting early (low volumes, down to 5 µL). The volume in one or more loops was then injected sequentially onto the ^2^D system, and finally detected using UV and MS. The ^2^D method served two purposes: providing further selectivity for oligonucleotide impurities; and, the main one, facilitating MS hyphenation by de‐salting the heart cut volume to remove phosphate buffer components. To accomplish the second purpose, the ^2^D method was required to both retain the oligonucleotides, as well as have a selectivity for the separation of phosphate salts and the oligonucleotides. This proved to be a non‐trivial task, since phosphates comprise much of the oligonucleotide backbone, and they share many characteristics. To this end several different stationary phases were investigated, mainly focusing on distinct RP or HILIC stationary phases.

Initially, HILIC stationary phases, exemplified with the Acquity BEH HILIC, was investigated for ^2^D purposes, the idea being that HILIC has previously been shown to have selectivity for oligonucleotides and related impurities [27], and using a different retention mechanism to the MMC ^1^D separation could provide further selectivity. However, although different selectivity and retention could be obtained on the Acquity BEH HILIC amide with e.g., a mobile phase of 10 mM ammonium Acetate pH 7 and a 5%–90% ACN gradient, compatibility with the ^1^D method was lacking and resulted in distorted chromatographic peaks, due to high water content in the injection medium for the ^2^D method.

Therefore, RP stationary phases, including the Acquity BEH C18, were investigated, the idea being that phosphate would have little to no retention, while some retention could be obtained for the oligonucleotides and related impurities. However, as mentioned above, since RP separations of oligonucleotides rely on ion‐pairing agents for retention, separation of the phosphates, and oligonucleotides on the RP phase was difficult to achieve, although it has been shown to be possible using MeOH and solvent modulation for desalting [16].

In the end, the Phenomenex LUNA Omega Sugar stationary phase provided necessary properties for the ^2^D. The LUNA Omega Sugar is designed for retention of highly polar compounds, especially carbohydrates and organic acids, and is generally considered a HILIC stationary phase (Phenomenex LUNA Omega SUGAR product information). The column chemistry comprises a mixture of amino and amide polyol groups with polar endcapping. Within the frame of the study, the column was initially considered for ^1^D oligonucleotide separations, but provided less selectivity than the Acclaim Mixed‐Mode RP/WAX‐1 column, e.g., using a mobile phase of 40 mM ammonium acetate buffer at pH 6.5 did not yield baseline separation of the main oligonucleotide and the larger impurities, the n‐1 and n‐2 short‐mers (data not shown). However, using a simple mobile phase gradient of 66.5%–19% ACN, the injected oligonucleotide was sufficiently retained on the column, with different selectivity for the phosphates, providing a means for separating these components. Furthermore, the final method provided enrichment of the oligonucleotide on the LUNA column, resulting in more than 100 times increased MS‐signal.

**FIGURE 7:**
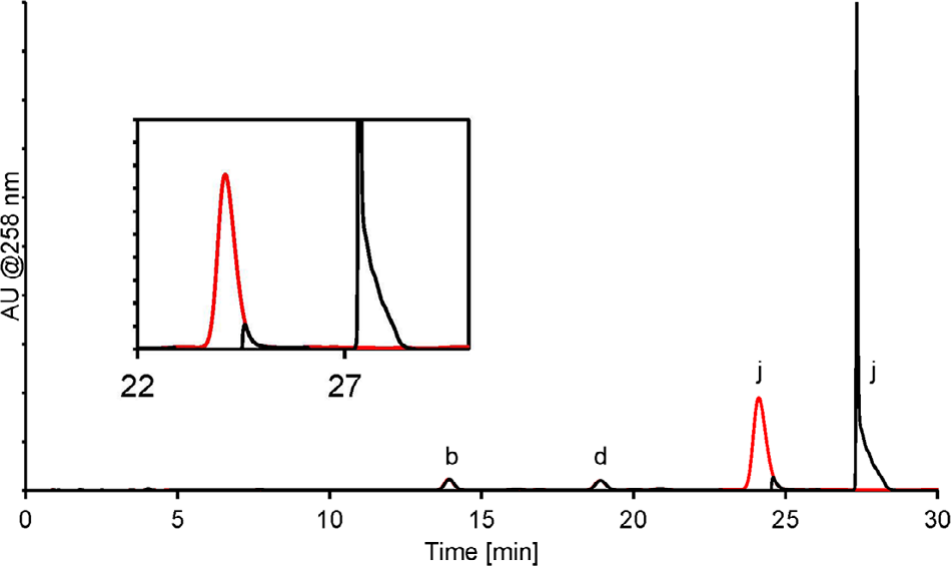
Overlay of UV‐traces from the (red) ^1^D method using Acclaim Mixed‐Mode RP/WAX‐1 column with ammonium phosphate buffer and 30% ACN, with 30 min pH  (7−8) and concentration (100−200 mM) gradient; and the (black) ^2^D method using a 250 µL loop and 1 min heart cut of the main oligonucleotide peak at 24 min injected on LUNA Omega SUGAR stationary phase with a 66.5%–19 % ACN gradient over 15 min in water. The cut‐out shows the zoomed‐in area around heart‐cut region. Peak labelling according to Table 1: (b) n‐2 short‐mer, (d) n‐1 short‐mer, (j) main 16‐mer oligonucleotide.

Although the LUNA stationary phase provided little extra selectivity for oligonucleotides and its related impurities, the combination of the separation properties of the MMC column in the ^1^D, and the de‐salting and enrichment properties of the LUNA column in the ^2^D provides the means to both separate, detect, and identify a large variety of oligonucleotides and impurities. Figure 7 shows the resulting chromatogram from the UV detection trace. The red signal corresponds to the ^1^D method described above; the black signal corresponds to the full 2DLC‐method. The cut‐out part of the main oligonucleotide peak at 24 min (Figure 7j) can be seen as a loss of signal during ca 1 min. The eluted peak from the 2D system can be seen at ca 28 min (Figure 7j). As shown, the resulting peak at 28 min have several characteristics deviating from a proper LC peak; it is rather a combination of two distinct parts. The sharp initial peak occurs because the oligonucleotides become concentrated on the ^2^D stationary phase, due to the weak eluting power of the medium. The broader, tailing part of the peak is caused by the system being overloaded with volume, which results in a longer release of the stored sample from the ^1^D of the separation process. This can be avoided by only injecting part of the stored volume, and discarding the rest to waste. However, the results shown in Figure 6 also illustrate the recovery of the heart‐cut peak, with low loss of sample due to the 2DLC method setup as evident by the peak areas at 24 and 28 min, respectively.

**FIGURE 8:**
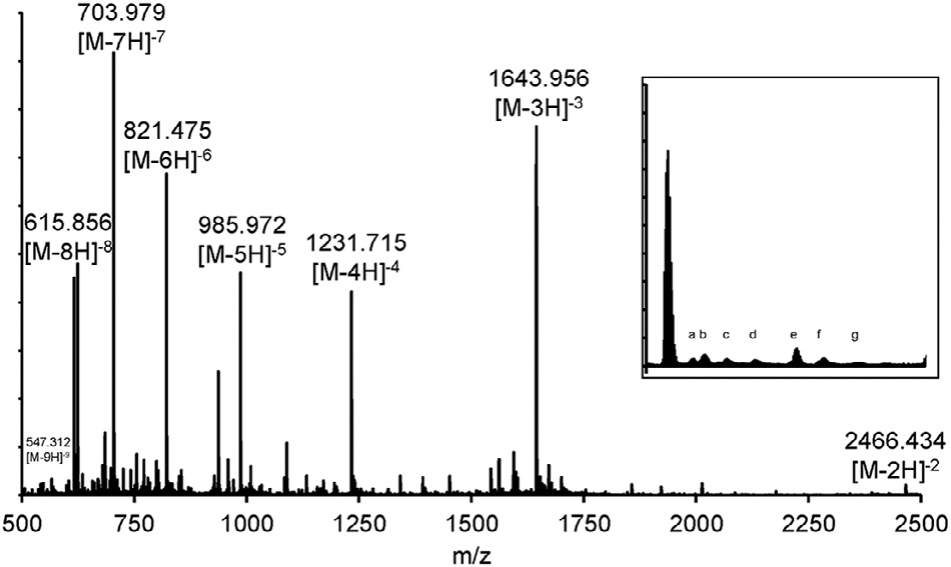
MS spectrum from heart cut main oligonucleotide peak at 28 min in previous Figure 7: ^1^D method using Acclaim Mixed‐Mode RP/WAX‐1 column with ammonium phosphate buffer and 30% ACN, with 30 min pH  (7−8) and concentration (100−200 mM) gradient; ^2^D method using a 250 µL loop and 1 min heart cut of the main oligonucleotide peak at 24 min injected on LUNA Omega SUGAR stationary phase with a 66.5%– 9% ACN gradient over 15 min. Letters in zoomed in section corresponds to (a) Na‐adduct, (b) K‐adduct, (c) Fe‐adduct, (d) ACN‐adduct, (e) P‐adduct, (f) P+Na‐adduct, (g) P+K‐adduct.

Figure 8 shows the resulting MS spectrum from the peak at 28 min, i.e., the main oligonucleotide. The pattern of several charge states for the oligonucleotide can be clearly seen, ranging from [M−2H]^−2^ to [M−8H]^−8^. The system utilized several loops, allowing the simultaneous identification of several components in an unknown sample. Table 1 contains MS‐acquired data on the peaks in the degraded oligonucleotide sample together with the suggested sequence. Since the focus of the study was on the separation method, proposed impurities/degradants structures are based on accurate mass measurement and deconvolution only at this stage. Future work would aim to confirm the proposed structure with further MS/MS fragmentation. Thus, the identities of the impurities in the table should be seen as tentative assignments. Still, note the range of characteristics for the various components, including long‐mers and short‐mers, as well as other degradation products like depurinated nucleobases and degraded nucleosides.

**TABLE 1:** Table with tentative sequences for peaks based on MS‐data. Labeling corresponding to labeling in Figure 2.

Retention time (min)	Theoretical monoisotopic mass (Da)	Experimental monoisotopic mass (Da)	Length; difference from main oligo (j)	Sequence (5′ → 3)
a) 12.45	4143.773	4143.727	14‐mer; (‐GGxA)	^HO^‐G xAA mCmCmC TTT GAmCT‐^OH^
b) 13.59	4276.812	4276.803	14‐mer; (‐GG)	^HO^‐G AAA mCmCmC TTT GAmCT‐^OH^
c) 16.65	4014.751	4014.722	13‐mer; (‐AmCT)	^HO^‐GGG AAA mCmCmC TTT G‐^OH^
d) 18.60	4605.864	4605.855	15‐mer; (‐G)	^HO^‐GG AAA mCmCmC TTT GAmCT‐^OH^
e) 19.76	3401.611	3401.579	11‐mer; (‐GGGAnA)	^PO4H2^‐A mCmCmC TTT GAmCT‐^OH^
f) 20.60	3714.668	3714.645	12‐mer; (‐GGGnA)	^PO4H2^‐AA mCmCmC TTT GAmCT‐^OH^
g) 20.60	4630.871	4630.812	15‐mer; (‐T)	^HO^‐GGG AAA mCmCmC TTT GAmC‐^OH^
h) 22.85	4027.726	4027.693	13‐mer; (‐GGnG)	^PO4H2^‐AAA mCmCmC TTT GAmCT‐^OH^
i) 22.85	4801.878	4801.843	16‐mer; (‐xA)	^HO^‐GGG xAA mCmCmC TTT GAmCT‐^OH^
j) 23.84	4934.917	4934.889	16‐mer; (main oligo)	^HO^‐GGG AAA mCmCmC TTT GAmCT‐^OH^
k) 25.51	4685.831	4685.769	15‐mer; (‐nG)	^PO4H2^‐GG AAA mCmCmC TTT GAmCT‐^OH^
l) 29.53	5263.969	5264.967	17‐mer; (+G)	^HO^‐GGGG AAA mCmCmC TTT GAmCT‐^OH^

*Note*: x = depurinated nucleobase, leaving an abasic site.

n = degraded nucleoside, leaving the phosphate group in the 5′‐position.

Finally, the zoomed in section of the MS spectrum in Figure 8 shows the typical adduct pattern of the oligonucleotides, in this case belonging to the [M−3H]^−3^ charge variant of the main oligonucleotide in the sample. The isotope and adduct formations of oligonucleotides are often complicated even further by co‐eluting impurities or ion‐pairing agents. However, by using the developed ^2^D method, the isotopes and adducts are limited to naturally occurring isotopes, like ^13^C, and mobile phase alkali metal residues (Na^+^, K^+^), simplifying the annotation. In addition, the ^2^D method can be developed further to allow the orthogonal separation of un‐resolved impurities. However, the current use of loops to store the eluent from the ^1^D limits the available choices of ^2^D methods, and the use of trap‐columns should be investigated as a further development.

## 4 Conclusions

The analysis of oligonucleotides and related impurities is a challenging task due to the variety of possible structural changes to the oligonucleotide as well as the large number of impurities with similar properties. Ongoing research cover development of new chromatographic methods for enhanced selectivity, as well as investigations into MS hyphenation of often unsuitable methods.

This study shows the successful implementation of MMC RP‐WAX separations of oligonucleotides and related impurities using UV detection, as well as the hyphenation to MS detection via 2DLC. The ^1^D method provides separations with high selectivity of oligonucleotides and related impurities, while the ^2^D provide MS compatibility and 100‐fold enhanced signal for detection of low concentration impurities. The full 2DLC method was subsequently used for the separation, detection and identification of the oligonucleotide, and degradation impurities.

## Author Contributions

**Jakob Haglöf**: Writing – Original Draft, Visualization, Supervision, Methodology, Formal Analysis, Conceptualization. **Jenny M. Nilsson**: Writing – Review & Editing, Visualization, Investigation, Formal Analysis. **Jufang Wu Ludvigsson**: Writing – Review & Editing, Supervision, Resources, Methodology, Conceptualization.

In the development of this work, the authors employed AstraZeneca in‐house ChatGPT tool to enhance readability and language quality. Following the utilization of this tool/service, the authors reviewed and edited the material as necessary and takes full responsibility for the content of the publication.

## Conflicts of Interest

The authors declare no conflicts of interest.
